# Ethics as if Your Life Depended on It

**DOI:** 10.1177/15562646261453477

**Published:** 2026-05-26

**Authors:** Joshua M. Pearce

**Affiliations:** 1 6221 Western University, London, Ontario, Canada

**Keywords:** Ethics, democratization, unintended consequences, innovation

## Abstract

**Purpose:**

This Commentary examines whether current ethics approval systems for medical research, grounded in the Belmont Report's principles of respect for persons, beneficence, and justice, paradoxically result in preventable deaths by delaying patient access to experimental therapies and proposes a framework for democratizing ethics oversight while maintaining safety.

**Findings:**

Peer-reviewed evidence demonstrates that ethics delays impose mortality costs that vastly exceed the harms they prevent, particularly for terminal illnesses. Bayesian decision analysis confirms current statistical thresholds are substantially more conservative than optimal for fatal diseases. COVID-19 pandemic responses proved that regulatory timelines can be dramatically compressed through organizational innovation, including accelerated vaccine development and distributed open-source manufacturing, without sacrificing safety.

**Conclusions:**

A five-part framework can enable patient autonomy in research ethics while preserving robust safeguards against exploitation: tiered consent based on disease severity, adaptive trial designs with patient governance, mandatory open-source transparency, post-market surveillance, and independent safety monitoring with patient representation.

## Introduction: The Paradox of Protection

There is a quiet catastrophe unfolding in the design of modern scientific medical research—one measured not in headlines but in absences: patients who never received a treatment, trials that never enrolled, therapies that arrived years too late. The ethics approval systems designed to protect human subjects have become, in many cases, the very mechanisms that condemn them. Because FDA-regulated clinical investigations cannot begin until they receive institutional review board (IRB) approval, lengthy and inconsistent IRB review processes, where local IRBs take months, delay and restrict clinical trials. This Commentary argues that the democratization of ethics approval, granting individuals meaningful authority over their own participation in experimental research, is a moral imperative. When the probability of a treatment's success is unknown, and the certainty of a disease's lethality is, the person whose life hangs in the balance must have a voice that the current system systematically denies them.

Modern research ethics rests on three principles articulated in the Belmont Report of 1979: respect for persons, beneficence, and justice (Protections (OHRP), 2018). These principles emerged from genuine horrors: the Tuskegee syphilis study, the Nazi experiments, and other episodes of unconscionable exploitation by researchers. No serious person disputes the necessity of ethical guardrails, but the Belmont Report itself identifies *respect for persons* as its first principle, that “individuals should be treated as autonomous agents” capable of “deliberation regarding their personal goals” (Protections (OHRP), 2018). The irony is that the regulatory apparatus built atop these principles has systematically undermined the very autonomy it was designed to protect, and the delays that result are now literally responsible for avoidable human deaths.

## The Lethal Cost of Delay

The evidence that regulatory delay slowed by ethics approvals kills people is empirical, quantifiable, and devastating. In his landmark 1973 analysis published in the *Journal of Political Economy*, economist Sam Peltzman evaluated the 1962 Kefauver-Harris Amendments, which ethically mandated proof of efficacy before drugs could reach the market (Peltzman, 1973). Peltzman found that the amendments had not measurably improved drug safety outcomes, but had dramatically slowed the introduction of new therapeutics, creating a net loss in consumer welfare. The cost of keeping beneficial drugs off the market (Type II error) can vastly exceed the cost of occasionally approving an ineffective one (Type I error), particularly for terminal illnesses (Isakov et al., 2019). For terminal illnesses like pancreatic cancer, the standard statistical significance threshold of 2.5% is substantially more conservative than the Bayesian Decision Analysis-optimal threshold of 23.9% to 27.8% (Isakov et al., 2019). Clearly, for these desperate patients, the cost of trying an ineffective drug is much less than the cost of not trying an effective one (Isakov et al., 2019).

We have known this for some time. As far back as 1974, Wardell documented what he called the “drug lag”: a systematic delay in the availability of new drugs in the U.S. compared with other developed nations (Wardell, 1974). Wardell demonstrated that drugs available for years in the United Kingdom and continental Europe were withheld from American patients for ethical reasons, and that this delay had concrete therapeutic consequences. Eichler et al. point out that regulatory systems are structurally biased toward visible harms (approving a bad drug) over invisible harms (withholding a good one), and call for reforms to better align regulatory risk acceptance with the interests of public health (Eichler et al., 2013). Mulligan's recent analysis confirms Peltzman's core finding using twenty-first-century data, showing that easing generic drug restrictions and accelerating vaccine approval during COVID-19 easily had net benefits that “more than offset” welfare costs (Mulligan, 2021). For every life saved by keeping bad drugs off the market, thousands are lost by keeping good drugs away from dying patients that would choose to try them if ethics approvals allowed for it.

The international drug lag compounds these losses and human suffering. Selecting one example of many around the world, Mo et al. found that among 242 orphan drugs approved by the FDA between 2013 and 2023, only 49.2% had been approved in China, with a median lag time of 1,004 days—nearly three years (Mo et al., 2025). For patients with rare and life-threatening diseases, three years is not a bureaucratic inconvenience; it is a death sentence. Other examples include: i) unequal access to newly registered cancer drugs across Europe resulted in an estimated loss of more than 30,000 life years for just two drugs (ipilimumab and abiraterone) due to delays in EMA approval relative to the FDA and subsequent national reimbursement barriers (Uyl-de Groot et al., 2020), ii) restrictive U.S. clozapine regulation and underuse likely contributed to avoidable mortality in treatment-resistant schizophrenia despite clozapine's mortality benefit (de Leon et al., 2025; Fernandez-Egea et al., 2024), and iii) the drug lag between Japan and the U.S. for anticancer drugs, with a median approval lag of 649 days for FDA-accelerated-approval oncology drugs, directly delays patient access to high-benefit therapies, particularly for rare cancers where the lag is worsening (Shiga et al., 2025; Taguchi et al., 2025).

## The COVID-19 Proof of Concept

If any event demonstrated that regulatory timelines based on current ethics rules are choices rather than immutable laws of nature, it was the COVID-19 pandemic. When it looked like we all might die, suddenly innovation trumped conventional regulations on two fronts.

First, Operation Warp Speed (OWS), a public-private partnership launched in May 2020, delivered multiple safe and effective vaccines within approximately ten months of the SARS-CoV-2 genetic sequence being open sourced, whichcompressed a process that typically takes ten to fifteen years (Winch et al., 2021). Although substantial resources were used and it relied on new technology, the key innovations were not scientific shortcuts but *organizational* ones (Winch et al., 2021). OWS's decisive breakthrough was organizational because money and technology only mattered once government restructured incentives, risk, and coordination; mRNA platforms, viral vectors, and protein subunits already existed before 2020, but OWS compressed years of sequencing, trials, manufacturing, and procurement into parallel rather than sequential steps by funding scale-up, harmonizing agencies, and guaranteeing demand through advance purchase commitments. It is argued here that this reduced commercial hesitation and manufacturing lag more than any single scientific discovery. In short, OWS's novelty was not inventing vaccine science, but building a wartime delivery system around it (Kuter et al., 2021). The National Academy of Medicine has affirmed that the vaccines authorized under Operation Warp Speed saved millions of lives and averted hundreds of billions of dollars in direct medical costs (NAM, 2025). A 2024 meta-analysis assessing 50 studies across four continents found these vaccines were 84–86% effective in preventing hospitalization (Wong & Mabbott, 2024).

Simultaneously, the ability to use digital fabrication technology to manufacture open source products in a distributed fashion was not only possible, but also faster and at lower costs (Pearce, 2020a). During the COVID-19 pandemic, the U.S. FDA relaxed enforcement policies and issued Emergency Use Authorizations to facilitate the rapid, local production of 3D-printed personal protective equipment (PPE), diagnostic swabs, and ventilator components. Rather than pass through the normal IRB and FDA approval process, the FDA collaborated with the NIH, VA and America Makes to create a curated collection of 3D-printed designs on the NIH 3D printing repository (https://3d.nih.gov/collections/covid-19-response). Open Source Medical Supplies organized over 74,000 makers, engineers, and designers to address severe shortages of PPE and medical devices. They documented delivery of over 48 million open-source products during the COVID-19 pandemic where they were most needed when conventional supply chains were crippled (*Open Source Medical Supplies*, 2026).

Operation Warp Speed and the distributed manufacturing of open source PPE and medical hardware during COVID-19 conclusively demonstrated that when the urgency is felt personally, the regulatory apparatus can move at a much faster pace without sacrificing safety. The uncomfortable corollary is that every year of “normal” research caused by ethics delays represents a political choice to tolerate preventable death. Here the golden rule provides clear moral guidance: do onto others as you would have them do onto you. If your life were on the line what would you want the ethical rules to be? You would want the choice to be yours.

## The Autonomy Argument

The case for democratizing research ethics approval begins with the Belmont Report's own first principle. Respect for persons demands that autonomous individuals be permitted to make informed decisions about risks to their own bodies (Protections (OHRP), 2018). The current system inverts this principle: an IRB, composed of professionals who do not have the disease in question, and who will not die if the research is delayed, makes binding decisions about what risks are “acceptable” for people who will.

Consider a patient with amyotrophic lateral sclerosis (ALS), a uniformly fatal neurodegenerative disease. The median survival from ALS diagnosis is two to five years. An experimental gene therapy shows promise in animal models but has not completed Phase III trials. Under the current system, an IRB may determine that the risks of the experimental therapy are too uncertain to justify a trial or may impose conditions that delay enrollment by months or years. The patient, whose alternative is certain death, has no meaningful say in this calculus. The IRB's assessment of “acceptable risk” is made from a position of safety that the patient does not share.

This is not a hypothetical injustice. It is the lived reality of millions of patients with terminal and serious illnesses. The U.S. Right to Try Act, signed into law in 2018, represented a partial acknowledgment of this problem by allowing terminally ill patients to access investigational drugs that have completed Phase I safety trials without FDA approval. But Right to Try is far too narrow in scope, applies only to drugs already in the pipeline, and does nothing to address the broader structural problem: that ethics review boards function as gatekeepers whose risk calculus systematically discounts the cost of inaction.

## A Framework for Democratic Ethics

Advocating for the democratization of ethics approval is not advocating for the abolition of ethical oversight. It is advocating for a system that takes its own foundational principles seriously. The following framework outlines how this could be achieved safely and ethically.

### First, Tiered Consent Based on Disease Severity

For patients with terminal diagnoses, the threshold for participation in experimental research should be set by the patient, not the board. The IRB's role should shift from gatekeeper to *information provider*: ensuring that the patient has open access to complete, clear, and comprehensible data about known risks, the current state of evidence, and the nature of uncertainty involved. The Belmont Report's requirements for informed consent: adequate information, comprehension, and voluntariness, are fully compatible with this model (Protections (OHRP), 2018). What changes is the locus of the final decision.

### Second, Real-Time Adaptive Trial Designs with Patient Governance

Modern statistical methods, including Bayesian adaptive designs, allow clinical trials to be modified in real time as data accumulates thereby expanding promising treatments, dropping futile ones, and adjusting dosages. These designs should be coupled with patient advisory councils. Patients enrolled in a trial have the most direct stake in its design and the most intimate knowledge of their own condition providing epistemological advantages.

### Third, Mandatory Transparency and Open Source Science

A common objection to expanded patient autonomy is that patients cannot evaluate complex scientific evidence. This objection has more to do with the current opacity of the research enterprise than with any inherent limitation of patients. For all publicly-funded research, the entire operation should be open source. While open access policies now mandate free readership of publicly-funded publications, taxpayers still cannot freely use the research they finance. All publicly-funded research outputs (e.g., software, hardware, drugs, etc.) should be open source by default, released under licenses permitting reuse, modification, and redistribution (Pearce, 2020b). Most importantly, for informed consent, all trial data (interim results, adverse events, protocol amendments) should be published in real time on publicly accessible platforms. This can help mitigate the risk that unscrupulous providers could market unproven treatments to desperate patients under the banner of “autonomy.” The informed consent process should include structured decision aids, developed in collaboration with patient advocacy organizations, which translate statistical uncertainty into comprehensible terms. Studies suggest that many potential participants can understand clinical trial information when it is written clearly and explained well. A Cochrane review found that enhanced consent materials - such as simplified forms, multimedia, and structured discussions - generally improve understanding compared with standard consent documents (Nishimura et al., 2013). Reviews in informed consent research similarly show that plain-language formats and teach-back methods improve comprehension, especially for complex risk/benefit information (Flory & Emanuel, 2004). Evidence supports the view that comprehension is often a presentation problem, not an inability of participants to understand. The goal is not to make every patient a biostatistician but to ensure that the information asymmetry between researchers and participants is minimized.

### Fourth, Post-Market Surveillance as a Substitute for pre-Market Delay

The COVID-19 experience demonstrated that robust Phase IV monitoring, tracking safety and efficacy after a treatment is in use, can substitute for years of pre-market evaluation without compromising patient safety (Winch et al., 2021). Japan's Regenerative Medicine Act already provides a model: conditional approval pathways allow promising therapies to reach patients while post-market data collection continues (*Act on the Safety of Regenerative Medicine - English - Japanese Law Translation*, n.d*.*). These therapies do not always end up with final approval (HeartSheet), but this approach recognizes that the perfect should not be the enemy of the good, and that data gathered from real-world use is often more informative than data from controlled artificial trial environments. This should not be interpreted as giving free-reign to pharmaceutical company marketing budgets, but rather a shift to rigorous and open post-market surveillance.

### Fifth, Independent Safety Monitoring with Patient Representation

Data Safety Monitoring Boards, which oversee ongoing trials and can halt them if safety signals emerge, should be expanded and should include patient representatives. These boards provide a critical safety net that is entirely compatible with expanded patient autonomy. The key distinction is between *stopping a trial because it is a net danger* (legitimate safety function) and *preventing a trial from starting because the outcome is uncertain* (paternalistic gatekeeping function). The former should be strengthened; the latter curtailed.

## Addressing the Objections

In the U.S. industry funds ∼70% of all clinical trials, but government funds the basic research behind nearly all newly approved drugs. For the latter acting in good faith, full transparency offers safeguards. For the former, critics will invoke the specter of exploitation: that desperate patients will be preyed upon by unscrupulous researchers at pharmaceutical companies. “Let the patient decide” sounds empowering until the patient is being informed by a company-sponsored decision aid. This concern is legitimate but misplaced as an argument against real democratization. Exploitation thrives in opacity, not in transparency. To counteract Type 1 errors caused by pharmaceutical companies using selective data presentation, dramatically strengthening the back-end accountability is necessary including mandatory real-time data disclosure, company-funded but *independently* administered surveillance, independent (non-sponsor) informed consent process, and enhanced liability for harms if data was incomplete or selectively presented. A system that is far more open source than it is now, that publishes *all* data in real time, requires structured informed consent, maintains independent safety monitoring, and empowers patient advisory councils is far more resistant to exploitation than the current system, in which IRBs operate behind closed doors, research is hidden behind intellectual property barriers, and patients have no formal voice.

In his influential 1969 essay Hans Jonas argued that society should never sacrifice research participants’ welfare for collective benefit, insisting on a descending order of permissibility that prioritized individual protection over social utility (Jonas, 1976). His position treats research participation as inherently exploitative and he thus demands near-absolute precaution. As is hopefully clear from the evidence provided in this commentary, this view is wrong because it ignores the moral cost of inaction: when regulatory caution delays life-saving drugs, patients outside trials may die preventable deaths. The ethical calculus must weigh harms to participants against harms to the broader population denied timely access. Accepting modestly increased participant risk to accelerate approval of demonstrably effective therapies reduces net suffering, which is a consequentialist argument that Jonas's deontological framework cannot accommodate. Schafer provided an initial critique (Schafer, 1983), but London (2021) delivered the most powerful criticism of Jonas's views for privileging symbolic protection of the few over measurable harm to the many. Refusing this tradeoff is itself an ethical choice, which may have lethal consequences.

Critics may also argue that such a path risks losing public trust in science (as more people will be harmed by research than they do now even if far more lives will be saved overall). The trust/loss objection is real but solvable. This trust objection is not that streamlined approval is inherently untrustworthy, it is that the opacity of the current system breeds distrust. Evidence shows trust in research is maintained or increased when participants receive transparent, comprehensible information and feel genuinely involved in decision-making (O’Neill, 2002). Real-time open data disclosure, independent safety monitoring, and patient advisory councils directly address trust concerns. Importantly, public trust eroded under the current restrictive system during COVID-19 (OECD, 2026), suggesting paternalism does not reliably produce trust. Transparency and genuine autonomy do. Research shows that public distrust stems not from participant harm per se but from opacity and perceived deception (Blom et al., 2025; O’Neill, 2002). Blom et al. (2025) demonstrate that adaptive consent, real-time data disclosure, and participatory governance actively build trust even in high-risk research contexts. Further, Kass et al. (1996) found that participants’ trust depends on honest communication, not on minimizing all risk. The proposed five-part framework directly addresses trust by making the system more honest, not less risky. Transparency about increased risk preserves trust; concealing the costs of delay destroys it.

Finally, critics will also argue that patients cannot assess risk rationally when facing a terminal diagnosis. But this argument proves too much. If the emotional weight of a terminal diagnosis disqualifies a patient from making decisions about experimental treatment, it should equally disqualify them from making decisions about palliative care, hospice enrollment, or the refusal of further treatment; all of which are currently protected as fundamental rights. The law already recognizes that competent adults may make consequential medical decisions under conditions of extreme duress. There is no principled reason to carve out an exception for research participation.

What would you want from research ethics approval systems if your life was on the line? We already know the answer as demonstrated by the successful approaches we took during COVID-19.

## Conclusions: The Moral Mathematics of Inaction

Every system of regulation embodies a set of moral priorities. The current ethics approval system prioritizes avoiding visible harm (the patient injured by an experimental treatment) over the prevention of invisible harm (the patient who dies waiting for a treatment that arrives too late). This asymmetry is not ethically neutral. It is a choice, and it is a choice that kills people.

The Belmont Report's principle of justice demands that the risks and benefits of research are distributed equitably (Protections (OHRP), 2018). There is nothing equitable about a system in which the costs of caution are borne entirely by the sick and dying, while the benefits of caution accrue to institutions and regulators who face no personal consequences for delay. Peltzman's analysis demonstrated this asymmetry empirically more than fifty years ago (Peltzman, 1973). The COVID-19 pandemic demonstrated that it can be overcome when the political will exists when ethics approval was made as if decision-makers’ lives were on the line. The question is whether we will extend that same urgency to the millions of patients with cancer, ALS, Alzheimer's disease, and rare genetic conditions who are dying today while ethics boards deliberate.

Democratizing research ethics approval does not mean abandoning research ethics. It means taking research ethics seriously enough to recognize that the greatest ethical failure is not the risk of an experiment gone wrong; it is the certainty of lives lost to the paralysis of institutional caution. The patient with a terminal diagnosis who wishes to try experimental therapy is not asking to be exploited. They are asking to be respected as an autonomous human being, capable of weighing uncertain hope against certain death, and choosing for themselves. That is not a radical demand. It is the Belmont Report's first principle, waiting to be honored.
